# A conserved hymenopteran-specific family of cytochrome P450s protects bee pollinators from toxic nectar alkaloids

**DOI:** 10.1126/sciadv.adg0885

**Published:** 2023-04-12

**Authors:** Julian Haas, Elena Beck, Bartlomiej J. Troczka, Angela Hayward, Gillian Hertlein, Marion Zaworra, Bettina Lueke, Benjamin Buer, Frank Maiwald, Michael E. Beck, Birgit Nebelsiek, Johannes Glaubitz, Chris Bass, Ralf Nauen

**Affiliations:** ^1^ Crop Science Division, Bayer AG, Alfred Nobel-Strasse 50, Monheim, Germany.; ^2^ University of Cologne, Department of Chemistry, Institute of Biochemistry, Cologne, Germany.; ^3^ College for Life and Environmental Sciences, University of Exeter, Penryn, Cornwall, UK.

## Abstract

Many plants produce chemical defense compounds as protection against antagonistic herbivores. However, how beneficial insects such as pollinators deal with the presence of these potentially toxic chemicals in nectar and pollen is poorly understood. Here, we characterize a conserved mechanism of plant secondary metabolite detoxification in the Hymenoptera, an order that contains numerous highly beneficial insects. Using phylogenetic and functional approaches, we show that the CYP336 family of cytochrome P450 enzymes detoxifies alkaloids, a group of potent natural insecticides, in honeybees and other hymenopteran species that diverged over 281 million years. We linked this function to an aspartic acid residue within the main access channel of CYP336 enzymes that is highly conserved within this P450 family. Together, these results provide detailed insights into the evolution of P450s as a key component of detoxification systems in hymenopteran species and reveal the molecular basis of adaptations arising from interactions between plants and beneficial insects.

## Teaser

A specific family of enzymes found in beneficial insects protects them from a plant defense chemical.

## INTRODUCTION

Plants have evolved sophisticated chemical defense strategies to protect themselves against herbivores and pathogens. These defenses rely on the production of plant secondary metabolites (PSMs), compounds that are not directly involved in primary physiological processes but enhance plant fitness to their environment (*1*). However, herbivores have, in turn, adapted to the presence of toxic PSM in their diet, for example, by evolving efficient detoxification strategies (*2*). This evolutionary arms race between plants and herbivores has fueled the emergence of key innovations such as PSM and detoxification systems of herbivores and is thought to have driven the diversification of both taxa (*3*–*5*).

Plant-herbivore interactions also affect species beyond those in direct competition, leading to potential evolutionary constraints on PSM production (*6*). Insect-pollinated plants, for instance, must balance the deterrence of herbivores with the attraction of insect pollinators. The influential “optimal defense theory” proposes that plant defense chemicals are concentrated in tissues that are essential for survival and most vulnerable to herbivory such as young leaves and reproductive structures (*7*, *8*). However, many plant species also produce floral rewards such as nectar or pollen in reproductive tissues; thus, insect pollinators can be exposed to PSM, including potentially toxic substances (*9*, *10*). Furthermore, multiple studies have shown a direct function for PSM in regulating pollinator visitation, e.g., by acting as deterrents or attractants, suggesting that they play an important ecological role in plant-pollinator interactions (*9*–*13*). Given this, it is likely that PSMs are a strong selective force on insect pollinators, potentially driving the evolution of detoxification mechanisms in a similar fashion to that reported for antagonistic insect herbivores (*14*). However, well-characterized examples of PSM detoxification in insect pollinators are rare (*15*, *16*).

Alkaloids comprise the most diverse group of PSM, with an estimate of over 25,000 compounds, and are especially abundant in angiosperms, which often rely on insect pollination (*17*). Studies of the honeybee *Apis mellifera,* one of the most ecologically and economically important generalist pollinator species, have demonstrated that many alkaloids are deterrent (*18*). Their acute toxicity, however, can vary substantially. The pyridine alkaloid nicotine, a potent natural insecticide, does not cause detrimental effects in honeybees, even after exposure to high concentrations for extended periods (*18*–*21*), making this species more tolerant to nicotine than many antagonistic herbivorous insects (*22*). Recent studies investigating the metabolic fate of nicotine in honeybees have linked this high tolerance to rapid and efficient metabolism in adults and larvae (*23*–*25*). While the major metabolic pathway seems to be cytochrome P450–mediated C′2-oxidation of the pyrrolidine ring (*24*), the key enzyme(s) involved remains unknown. Furthermore, whether alkaloids are metabolized by P450s in other Hymenoptera, the most important insect order of plant pollinators, has not been established.

In the present study, we used a recently described toxicogenomics approach (*26*–*28*) to gain insight into several outstanding questions relating to the adaptation of insect pollinators to PSM, including the following: (i) How do insect pollinators detoxify PSM, as exemplified by detoxification of nicotine and other alkaloids? (ii) To what extent are the underpinning molecular mechanism(s) of alkaloid detoxification conserved across the Hymenoptera? (iii) Can we identify critical structural and functional determinants of PSM metabolism (e.g., amino acids in enzymes that are key determinants of alkaloid metabolism), and what evidence is there that these features are evolutionary conserved in species that can detoxify alkaloids?

## RESULTS

### Functional expression of the entire honeybee CYP3 clan identifies CYP336A1 as the key determinant of nicotine metabolism

**Fig. 1.**
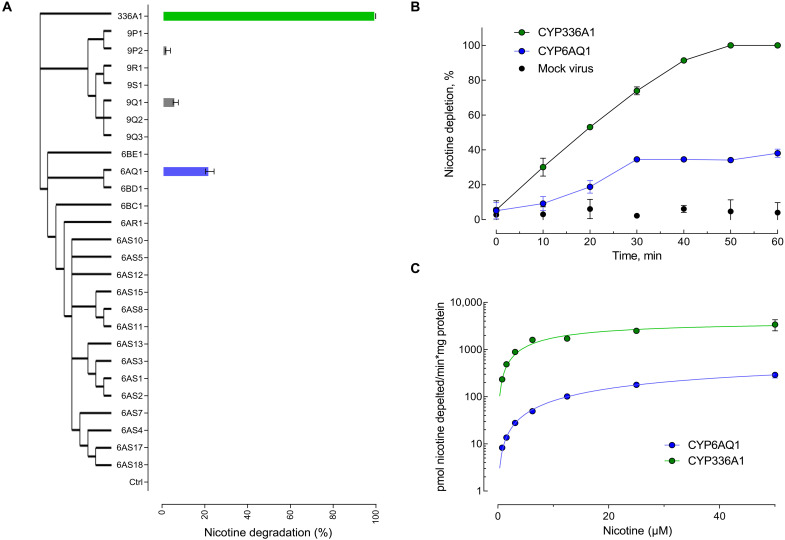
Nicotine depletion by clade 3 cytochrome P450s of the honeybee. (**A**) Nicotine degradation by functionally expressed cytochrome P450 analyzed by UPLC-MS/MS (data are mean values ± SD, *n* = 3). Branches display phylogeny. (**B**) Time dependence of nicotine depletion mediated by CYP336A1 (green) and CYP6AQ1 (blue). (**C**) Michaelis-Menten kinetics of nicotine depletion by recombinantly expressed CYP336A1 and CYP6AQ1. Error bars display SD (*n* = 3).

We first focused on characterizing the molecular basis of honeybee tolerance to the iconic alkaloid nicotine. As previous research has implicated metabolism of this PSM by honeybee P450s (*23*–*25*), we conducted an ambitious functional analysis of this enzyme superfamily by recombinantly expressing 27 of the 46 honeybee P450 genes, comprising the entire CYP3 clade. We focused on P450s in this clade as its members have been most frequently linked to xenobiotic detoxification across a range of insect species (*29*). When these enzymes were individually screened for nicotine metabolism using ultraperformance liquid chromatography–tandem mass spectrometry (UPLC-MS/MS), just four P450s exhibited a degree of activity against this compound. Two P450s, CYP9P2 and CYP9Q1, showed basal activity with 2.8 and 6.4% depletion, respectively. CYP6AQ1 exhibited greater activity with an average of 22% depletion, while CYP336A1 depleted 10 μM nicotine entirely (i.e., 100% depletion) (Fig. 1A). Time dependency studies revealed a linear increase of nicotine degradation for CYP336A1, resulting in complete depletion of 10 μM nicotine after 50 min (Fig. 1B). In contrast, CYP6AQ1-mediated nicotine degradation increased linearly for 30 min, but at a lower rate, with metabolism ceasing after this time point. Michaelis-Menten kinetics confirmed the predominant role of CYP336A1 in nicotine metabolism with a *V*_max_ of 4024 pmol nicotine metabolized/min per mg protein (~5-fold higher than CYP6AQ1) and a Michaelis constant (*K*_m_) value of 12.77 μM (~7-fold lower than CYP6AQ1) (Fig. 1C).

To further explore the role of CYP336A in xenobiotic detoxification in vivo, we analyzed its expression level in different life stages and tissues of the honeybee via quantitative polymerase chain reaction (qPCR). CYP336A1 was found to be highly expressed in late larval instars and adult honeybees. In adult honeybees, its expression was especially high in Malpighian tubules and the brain, key sites of xenobiotic detoxification and the neuronal receptor of nicotine (the nicotinic acetylcholine receptor), respectively (fig. S1). Together, these results provide unequivocal evidence of the key role of CYP336A1 in nicotine metabolism.

### CYP336A1-mediated oxidative dehydrogenation is the first step of nicotine degradation in honeybees

**Fig. 2.**
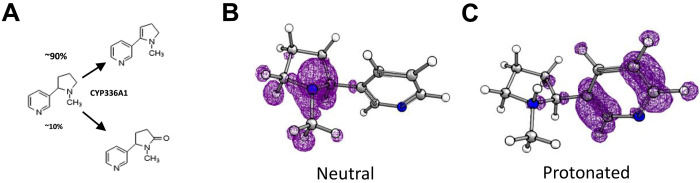
Identification of metabolites resulting from nicotine breakdown by CYP336A1. (**A**) Formation of *N*-methylmyosmine (90%) and cotinine (10%) as major metabolites after incubation of CYP336A1 with nicotine. Fukui functions of neutral (**B**) and deprotonated (**C**) nicotine showing different function maxima. Under the experimental conditions (pH 7.6), most of the nicotine is assumed to be protonated, suggesting the C′2 atom of the pyrrolidine ring as a major potential site for an electrophilic attack.

Next, we aimed to identify the metabolite(s) resulting from oxidative metabolism of nicotine by CYP336A1. In vivo, several metabolites have been previously identified, with 4-hydroxy-4-(3-pyridyl) butanoic acid, the final product of the C′2 oxidation pathway, the major metabolite (fig. S2) (*24*). However, we could not identify this metabolite or its keto acid/aminoketone precursors after incubating CYP336A1 with nicotine. Rather, two metabolites with mass-to-charge ratio (*m/z*) 160 (M-2) and 176 (M+14) were identified. By using commercial reference standards, we confirmed that the M+14 metabolite corresponds to cotinine. Subsequent quantification revealed that only approximately 10% of nicotine is transformed to cotinine, while the majority is converted to the M-2 metabolite. On the basis of the literature, there were two strong candidates for the M-2 metabolite: *N*-methylmyosmine (tautomer of nicotine-Δ^1′(2′)^ iminium ion (*30*) or nicotine-Δ^1′(5′)^ iminium ion (*31*, *32*). *N*-methylmyosmine could be sourced as a reference standard, and UPLC-MS/MS analysis provided clear evidence that the M-2 metabolite was indeed this compound (Fig. 2A). Thus, we propose that *N*-methylmyosmine is the first product of the nicotine C′2 oxidation pathway in honeybees mediated by CYP336A1.

To support this conclusion, Fukui functions *f*^−^(***r***) for attack by an electrophile were calculated. Maxima of *f*^−^(***r***) correspond to regions in space, where the electron density of molecules is most likely to be reduced and thus, in many cases, coincide with sites of metabolic attack by oxidative agents, including P450s (*33*). Nicotine is a dibasic compound with a p*K*_a_ (where *K*_a_ is the acid dissociation constant) value of 8.1 for the pyrrolidine nitrogen (*34*). Under physiological [pH in honeybee fluids: 6 to 7 (*35*, *36*)] and experimental conditions (pH 7.6), most of the pyrrolidine nitrogen are assumed to be protonated. This is relevant as the local reactivity pattern markedly changes between the two forms as evident by the respective Fukui functions (Fig. 2, B and C). The neutral form exhibits maxima for an electrophilic attack (e.g., by P450s) across the *N*-methyl-pyrrolidine ring corresponding to sites of major nicotine metabolites such as cotinine (5′C-oxidation), nicotine *N*-oxide (*N*-oxidation), or nornicotine (*N*-demethylation). In contrast, the Fukui function of protonated nicotine exhibits a high electron density across the pyridine moiety and only minor areas within the pyrrolidine ring surrounding the C′2 atom. Together, these results support the hypothesis that nicotine is metabolized by CYP336A1 in its protonated form by oxidative dehydrogenation at the C′2 atom of the pyrrolidine moiety (fig. S2).

### CYP336A1 substrate profiling shows specific activity toward pyridine and tropane alkaloids

Following the identification of CYP336A1 as the major metabolizer of nicotine in honeybees, we investigated the broader substrate profile of this P450. CYP336A1 was initially screened against 13 model substrates routinely used in P450 research, comprising seven model substrates derived from coumarin, five model substrates derived from resorufin, and *p*-nitroanisole. CYP336A1 exhibited no notable metabolism of any model substrates in this initial panel (table S1). Given its established metabolic activity for nicotine, we widened our substrate panel to explore whether CYP336A1 exhibits specific activity toward additional alkaloids and extended our analysis to include anabasine, atropine, scopolamine, cytisine, and echimidine. From a functional perspective, these substances share the ability to bind to nicotinic or muscarinic acetylcholine receptors, respectively (*37*–*39*). Structurally, they all contain a basic nitrogen, and their protonation status changes their reactivity pattern (based on *f*^−^ maxima of the respective Fukui function) (fig. S3). We also included the nonbasic alkaloid caffeine as well as coumarin in our investigation as these two phytochemicals have pharmacologically different actions and do not have a basic nitrogen.

**Fig. 3.**
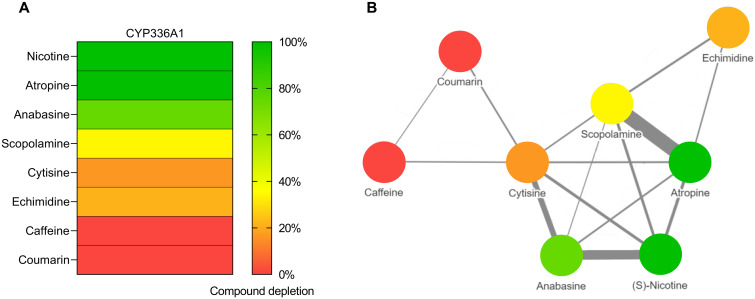
Substrate profile of honeybee CYP336A1. (**A**) Metabolism of selected phytochemicals by functionally expressed CYP336A1. Color represents the extent of observed depletion (increasing from red to green). (**B**) Chemical similarity network calculated by Morgan fingerprints showing similarities exceeding 30%. Thickness of connecting lines correlates with degree of similarity.

Incubation of CYP336A1 with the selected set of alkaloids revealed differential metabolic activity for different compounds (Fig. 3A). The pyridine alkaloid anabasine (74.5% depletion ± 1.2% SD) and the tropane alkaloid atropine (99% ± 0.23%) were rapidly metabolized. CYP336A1 was also able to degrade scopolamine (34.4% ± 1.46%), echimidine (21.4% ± 3.26%), and cytisine (16.6% ± 5.13%); however, there was no activity toward caffeine or coumarin. To gain insight into structure-activity relationships, a similarity network of the molecular structures of the eight phytochemicals was calculated using a cheminformatic approach (Fig. 3B). Within the similarity network, nicotine, atropine, and anabasine, for which CYP336A1 activity was highest, are the least similar to caffeine and coumarin, the compounds that were not metabolized by CYP336A1. Thus, even for this relatively small set of compounds, a structure-activity relationship was apparent, with the substrate range of CYP336A1 centered around alkaloids containing a basic nitrogen.

### Genomic analysis reveals the conservation of CYP336 genes across the order Hymenoptera despite intrachromosomal relocation

**Fig. 4.**
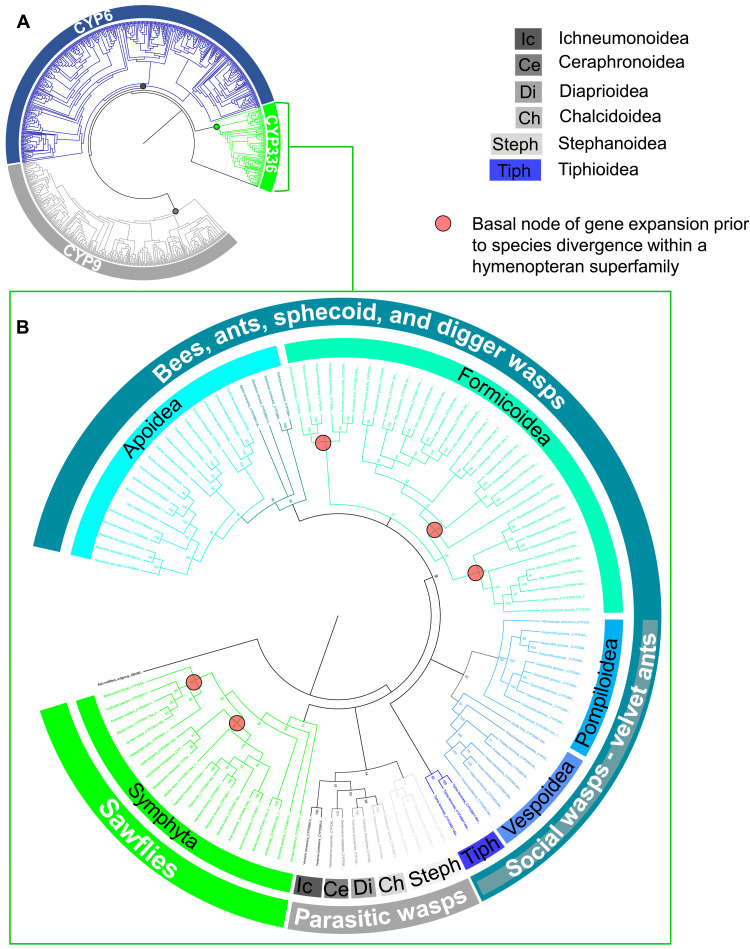
Phylogenetic analysis of the evolutionary origin of the CYP336 family of P450s through the Hymenoptera. (**A**) Phylogeny of the CYP3 clan of P450s from 40 hymenopteran species showing the three major gene families: CYP6 (blue), CYP9 (gray), and CYP336A (green). (**B**) Phylogenetic relationship of the CYP336 family. P450s were aligned with the outgroup *A. mellifera* CYP314A1 (GB45651) in Geneious version 10.2.6 (Biomatters) using MUSCLE (version 3.5). Amino acid substitution was modeled using the LG replacement matrix (*87*). Phylogeny estimated: (A) maximum likelihood algorithm; (B) Bayesian inference (chain length, 1,100,000; subsampling frequency, 200; burn-in length, 100,000; heated chains, 4; heated chain temperature, 0.2). Nodes where gene duplication occurs before species divergence are marked with a red circle. Abbreviated wasp superfamily names: Ic, Ichneumonoidea; Ce, Ceraphronoidea; Di, Diaprioidea; Ch, Chalcidoidea; Steph, Stephanoidea; Tiph, Tiphioidea.

Recent studies have shown functional overlap of evolutionary-related P450s involved in the metabolism of synthetic insecticides across the diversity of bee pollinators (*28*, *40*). To investigate whether different hymenopteran species may recruit evolutionarily-related mechanisms to metabolize PSM such as alkaloids, we analyzed the evolutionary origin of CYP336A1 by phylogenetic analysis of the CYP3 clan of P450 enzymes of 48 hymenopteran species (table S2). In total, 110 CYP336 genes were identified in all major hymenopteran lineages encompassing sawflies, wasps, ants, and bees. The CYP336 branch forms a sister group to the remaining members of the CYP3 clan (CYP9 and CYP6 families) and may be viewed as the basal branch of the clan (Fig. 4A). In most species (45 of 48), at least one member of the CYP336 family could be identified, with up to nine genes found in the ant *Dinoponera quadriceps*. Furthermore, interrogation of the CYPome of insects from other orders available on the National Center for Biotechnology Information (NCBI) failed to identify a named CYP336 gene, suggesting that this P450 family is specific to the Hymenoptera.

A focused phylogenetic analysis of the CYP336 family of P450s was performed to further elucidate the evolutionary relationship within this lineage of genes (Fig. 4B). Most nodes in the tree had strong Bayesian posterior probability support (>80%). As expected, the sawflies (Symphyta) are basal to the phylogeny. Most sawfly species have a single CYP336 gene (12 of 17); however, in certain members of the largest superfamily, the Tenthredinoidea, there has been an expansion of the CYP336 family. *Neodiprion lecontei, Tenthredo koehleri*, and *Athalia rosae* have two, three, and five sequences, respectively, and there is possible evidence of a duplication event before its divergence from other Tenthredinoidea species in *A. rosae* (basal nodes marked with a red circle in Fig. 4B). It also appears that the ancestor of the Formicoidea had at least two, and perhaps three, CYP336 genes (Fig. 4B). Thus, it seems likely that the ancestral “proto-ant” had an expanded repertoire of this lineage. Where there have been duplications and expansions of the CYP336 family in the other Hymenopteran superfamilies, they appear to have occurred after the divergence of genera. In summary, while these analyses uncover variation in CYP336 gene number between lineages, they reveal a remarkable level of conservation in CYP336 gene presence across the diversity of Hymenoptera, suggestive of strong selection acting to maintain gene presence.

To further explore the evolution of the CYP336 family within the Hymenoptera, we assessed the conservation of the genomic region harboring these genes. To look for evidence of macrosynteny, chromosomes containing *CYP336* sequences were aligned in pairwise comparisons and as one multiple chromosomal alignment (fig. S4). With the exception of chromosome 25 in *Vespa crabro* [~3.6 million base pairs (Mbp)], all chromosomes were >15 Mbp long and the pairwise comparisons to *N. lecontei* reveal a pattern of decreasing numbers of locally collinear blocks (LCBs) with evolutionary distance (table S3). For example, there are 100 LCBs in the comparison of *N. lecontei* (Symphyta) and *Venturia canescens* (Ichneumonoidea) chromosomes, but only 59 in the alignment to *A. mellifera* (Apoidea). The apparent lack of syntenic relationship in *V. crabro* (17 LCBs) is likely to be explained by the difference in the size of the chromosome containing the *CYP336* genes in this species.

**Fig. 5.**
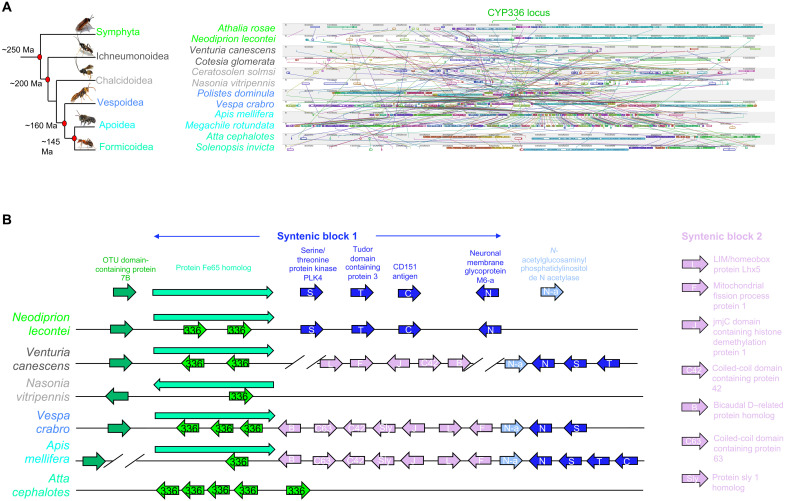
Syntenic relationship of the CYP336 loci in 12 hymenopteran species from six superfamilies. (**A**) LCBs identified at the CYP336 loci in 12 species from six hymenopteran superfamilies. Each colored shape is a region without rearrangement of homologous backbone sequence (a collinear block). Lines between sequences trace orthologous LCBs through the genomes. (**B**) Schematic representation of the syntenic relationship at the CYP336 loci in six hymenopteran species across six superfamilies. Arrows represent syntenic genes and denote reading frame (not drawn to scale).

To assess the level of conserved microsynteny, the region 500 kbp upstream and downstream of the *CYP336* genes was interrogated and flanking genes were identified (data S1). When intra-superfamily relationship is considered, the genomic region appears highly uniform with extensive LCBs and conserved gene order within the Symphyta, Vespoidea, Formicoidea, and Apoidea (Fig. 5A, fig. S5, and data S1). There is less evidence for conserved microsynteny in the parasitic wasps, particularly within the Ichneumonoidea superfamily (fig. S5 and data S1).

Comparing the genomic region across the Hymenoptera superfamilies, it appears that there is a pattern of intrachromosomal shuffling of syntenic blocks of genes rather than interchromosomal rearrangement (data S1). With the exception of the Formicoidea and parasitic wasps, a syntenic block (SB1) containing five genes (*protein Fe65 homolog*, *serine/threonine protein kinase PLK4*, *tudor domain-containing protein 3*, *CD151 antigen*, and *neuronal membrane glycoprotein M6-a*) is found in close association to the *CYP336* genes (Fig. 5B and data S1). However, in the Ichneumonoidea, SB1 is found on the same chromosome as the *CYP336* genes, but not in close association with them. A second syntenic block (SB2) composed of five to seven genes, found on a separate chromosome in the sawflies, has been inserted into SB1 in the Vespoidea and Apoidea (Fig. 5B). SB2 is also found in close proximity to SB1 in the Ichneumonoidea (data S1). In the Chalcidoidea, most of the genes making up both syntenic blocks are located on separate chromosomes/scaffolds to the *CYP336* genes (data S1), indicating an interchromosomal rearrangement in this superfamily, which likely occurred after the divergence of the Aculeata, approximately 160 million years (Ma) ago. However, the genes framing the *CYP336* sequences are identical to those found in sawflies and the other wasp superfamilies (*protein Fe65 homolog* and *OTU domain-containing protein 7B*) (Fig. 5B and data S1). The CYP336A locus in the Apoidea is framed by *protein Fe65 homolog*, SB1, and SB2, but, in *A. mellifera*, *OTU domain-containing protein 7B* is located 2 Mbp away (data S1).

Ants appear to be the only superfamily that shows no conserved syntenic relationship around the CYP336 locus. Both SB1 and SB2 appear together on a different chromosome, indicating that the rearrangement of the CYP336 locus may have occurred after divergence from the other Hymenopteran superfamilies but before the separation of the ants into their subsequent families approximately 145 Ma (Fig. 5A). Since the largest expansion of this lineage of P450s has occurred in ants, it may be that the translocation of this locus is linked to the duplication events that have occurred.

In summary, much of the global gene content of the chromosome containing the CYP336 locus has been conserved over the long time scale of Hymenoptera evolution from sawflies to bees, albeit with intrachromosomal translocations and inversions. There is less evidence for interchromosomal translocations across the order. Our findings contrast with previous syntenic analysis of P450 clusters in Lepidopteran species that reported a high number of syntenic breaks, evidence of frequent chromosomal rearrangements, and a lack of microsynteny as evidenced by marked changes in flanking genes (*41*). Together, the patterns observed in our study suggest selection acting to conserve synteny at the CYP336 locus and may reflect the requirement to maintain sets of locally adapted genes.

### Recombinant expression of CYP336A1 functional orthologs confirms a shared alkaloid substrate profile

**Fig. 6.**
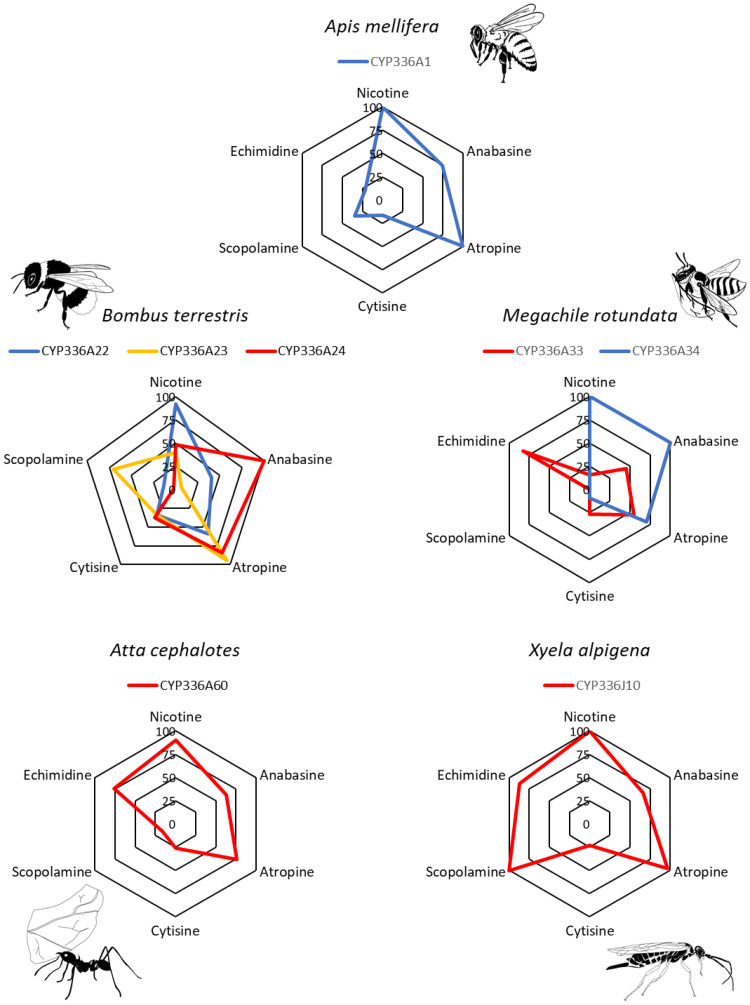
Functional expression and investigation of alkaloid metabolism by eight members of the CYP336 family from five representative hymenopteran species. Spider plots show depletion (%) compared to control samples without NADPH regeneration system (*n* = 3). Paralogs of the same species are illustrated in the same plot with different colors.

To investigate whether there is evidence of conserved function within the CYP336 family in terms of activity against alkaloids, seven genes from four representative hymenopteran species that diverged over 281 Ma (*42*) were selected for functional expression. These comprised three CYP336 family P450s from the bumble bee *Bombus terrestris*, two genes from the alfalfa leaf-cutting bee *Megachile rotundata*, one gene from the leaf-cutting ant *Atta cephalotes*, and one gene from the plant-parasitic sawfly *Xyela alpigena* (table S4). Incubation of the recombinant CYP336A proteins with alkaloids containing a basic nitrogen revealed a conserved capacity to metabolize these phytochemicals (Fig. 6 and table S5). Greatest activity was seen for nicotine, anabasine, and atropine across P450s of the four species (90.46 to 100%, 62.86 to 100%, and 70.5 to 96.78% depletion, respectively), while cytisine was metabolized least efficiently (<38% depletion). Despite this conserved capacity to metabolize alkaloids, substrate preferences differed substantially across the seven CYP336A proteins, especially for closely related enzymes within species. For example, in *B. terrestris*, CYP336A22 shows efficient metabolism of nicotine and atropine (92.15 and 59.34% depletion, respectively) but is less active against the other alkaloids screened (12.94 to 41.05% depletion). In contrast, CYP336A23 was particularly active against the tropane alkaloids atropine and scopolamine (94.1 and 70.2% depletion, respectively), and CYP336A24 metabolized anabasine most efficiently (99.97% depletion) (table S5). Similarly, *M. rotundata* CYP336A34 exhibited high activity against the pyridine alkaloids nicotine and anabasine (100% depletion) but was unable to metabolize echimidine. CYP336A33 was less active against the pyridine alkaloids (<45% depletion) but showed efficient metabolism of echimidine (82.57% depletion). To gain further insight into the potential divergence of paralogous *CYP336A* genes, we examined the expression of *CYP336A* isoforms in the bumblebee and alfalfa leaf-cutting bee. This revealed that paralogous *CYP336A* genes in these species are divergently expressed in different tissues or body segments (fig. S6). In combination with our functional analyses, this suggests that *CYP336A* gene duplication events have led to partial subfunctionalization of paralogs coupled with further differentiation in terms of their tissue-specific expression patterns.

### The conserved alkaloid-metabolizing capacity of the CYP336 family is associated with an unusual aspartic acid residue in the cytochrome P450 I-helix

The remarkable level of functional conservation observed across the Hymenopteran species investigated in terms of CYP336A activity against basic alkaloids offers an exceptional opportunity to identify key structure-function determinants of P450-mediated metabolism of a group of model PSM. To investigate candidate amino acid residues that might be key to this conserved function, we used the SDPlight algorithm (*43*). Five amino acids were scored as different between the CYP336 family and the remaining CYP3 clan enzymes (table S6). Four of these five residues are located in the P450 substrate recognition sites defined by Gotoh (*44*), suggesting a potential role in substrate recognition.

**Fig. 7.**
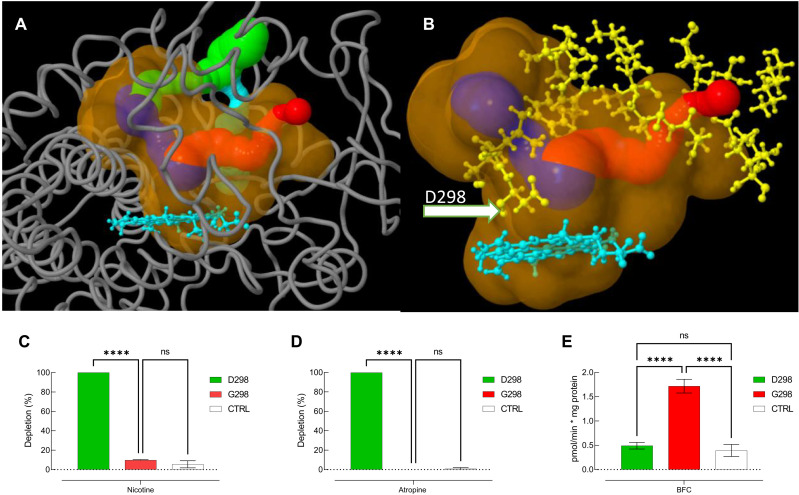
Computational 3D modeling and site-directed mutagenesis of *A. mellifera* CYP336A1. (**A**) 3D model of CYP336A1 generated using AlphaFold2. The heme group is colored cyan. The catalytic pocket is colored in orange, and the calculated access channel is colored in blue (most relevant channel), red (second relevant channel), and green and cyan (extensions of the other two channels). (**B**) Close-up view of the catalytic pocket and the two relevant access channels, with an arrow pointing at the conserved aspartic acid residue. The residues contributing to the bottlenecks of the access channels are shown in yellow. Substitution of the aspartic acid residue within the I-helix with a glycine (D298G) results in the loss of function of nicotine (**C**) and atropine (**D**) metabolism. (**E**) Metabolic capacity toward 7-benzyloxy-4-(trifluoromethyl)coumarin (BFC) is activated after introduction of the D298G mutation. Data are mean values ± SD (*n* = 3). Asterisks indicate significant differences between enzymes (one-way ANOVA, *P* < 0.0001; ns, not significant).

We extended this analysis by generating a three-dimensional computational model of CYP336A1 using AlphaFold2 (*45*, *46*). This resulted in a high-quality model based on an ERRAT score of >90% and a Ramachandran plot showing only one amino acid in a disallowed region (Ile^41^; fig. S7). Using CAVER Web v1.1 (*47*), we analyzed the protein model with regard to the catalytic pocket and access channels. One major catalytic pocket surrounding the heme prosthetic group with a volume of 1464 Å was identified, and four access channels were detected, with two of them being an extension of the other two (Fig. 7A). The most relevant channel (based on throughput probability and lowest necessary activation energy; fig. S8) has a total length of 13.3 Å and a bottleneck radius of 1.4 Å. The bottleneck is framed by seven amino acid residues (fig. S8). When comparing those residues with the CYP336-specific residues identified using SDPlight, only one residue is common to both analyses: an aspartic acid residue (D298). This residue is located at the end of the identified bottleneck and directly opposite the heme prosthetic group within the I-helix of CYP336A1 (Fig. 7B). Furthermore, D298 is highly conserved in CYP336 enzymes across the Hymenopteran species included in the earlier phylogeny (fig. S9), while the remaining CYP3 clan P450s usually have an alanine or glycine at the corresponding position. Given its location and its intrinsic properties (a negatively charged COO^−^ side chain under physiological conditions), we hypothesize that D298 functions as a “gatekeeper” by facilitating access and binding of protonated substrates such as alkaloids within the catalytic pocket of CYP336 genes.

To validate these predictions, we used site-directed mutagenesis to substitute the aspartic acid with glycine in *A. mellifera* CYP336A1 and incubated the recombinant mutant P450 with nicotine and atropine. After introduction of the D298G mutation, the metabolic activity of CYP336A1 toward nicotine and atropine was completely abolished with no significant difference in depletion observed compared to the negative control (Fig. 7, C and D). We observed an opposite effect for the model substrate 7-benzyloxy-4-(trifluoromethyl)coumarin (BFC; a nonbasic coumarin derivative). While CYP336A1 does not metabolize BFC to any measurable extent, the D298G substitution leads to significant activity toward BFC (Fig. 7E). Together, these results reveal that the highly conserved D298 is a signature amino acid for the CYP336 family in Hymenoptera and a key determinant of the specific activity of this P450 family toward protonated substrates such as alkaloids.

## DISCUSSION

PSMs are ubiquitous across the plant kingdom and play a well-established role in protecting plants from antagonistic insect herbivores. However, the presence of PSM in the nectar and pollen of angiosperms has also been proposed to drive the coevolution of plants and beneficial insects such as pollinators (*11*). Here, we provide evidence in support of this hypothesis by uncovering a conserved mechanism of PSM detoxification in the Hymenoptera.

Using the alkaloid nicotine and the honeybee as our initial model, our analyses identified cytochrome P450s, and specifically CYP336A1, as highly efficient metabolizers of alkaloids in this species. The cytochrome P450 superfamily plays a prominent role in the detoxification of phytochemicals in herbivorous insects (*48*). However, a growing body of work has also provided evidence of the importance of this enzyme superfamily in xenobiotic detoxification in beneficial insects such as bees. For example, members of the CYP6 and CYP9 families of P450s have been linked to the metabolism of both phytochemicals such as flavonoids and several synthetic insecticides (*15*, *26*, *27*, *49*, *50*). Our findings on the specific activity of CYP336A1 against alkaloids containing a basic nitrogen extend the repertoire of known xenobiotic metabolizing P450s in bees and further illustrates the essential role of the honeybee CYPome in environmental response. The important role of CYP336A1 in xenobiotic metabolism is further supported by our analysis of the spatiotemporal mRNA expression pattern of this P450 in biologically relevant tissues and life stages. Most notably, the high levels of expression of *CYP336A1* in the Malpighian tubules observed in our study are consistent with previous evidence, suggesting that these organs are able to efficiently remove nicotine from the hemolymph and metabolize it in honeybees (*51*). Together with previous work (*26*), these findings suggest that the Malpighian tubules are a key site of xenobiotic metabolism in bees (*24*).

Previous in vivo research on the metabolic fate of nectar nicotine in honeybees implicated metabolism of this compound by 2′C-oxidation (*24*). Our analysis of the metabolites resulting from oxidative metabolism of nicotine by CYP336A1 is consistent with this and suggests that CYP336A1 catalyzes the first step of the C′2 oxidation pathway by converting nicotine to *N*-methylmyosmine. A similar route of nicotine mechanism has been previously reported for the P450 CYP6CY3 in the aphid *Myzus persicae* (*31*) and CYP2A6 of humans (*32*). The subsequent conversion of *N*-methylmyosmine to the corresponding aminoketone may be driven by spontaneous rather than enzymatic hydrolysis (*52*). Further research is warranted to identify the enzymes catalyzing the subsequent reactions resulting in 4-hydroxy-4-(3-pyridyl) butanoic acid in honeybees (*24*). In lepidopteran species, such as the tobacco hornworm *Manduca sexta* (a specialist herbivore of tobacco) or the cabbage looper *Trichoplusia ni* (a generalist herbivore), a different P450-mediated biotransformation pathway is predominant. Here, *N*-oxidation and 5′C oxidation have been reported as the major metabolic pathways (*53*, *54*). One notable feature of many lepidopteran species is their alkaline gut environment with pH values up to 12 in the midgut where nicotine metabolism in lepidopteran species is assumed to be initiated (*55*). These high pH values favor the occurrence of nicotine in its neutral form. In this regard, Fukui functions suggest that only nicotine in its neutral form is accessible to electrophilic attack at the 5′C or the *N*-methyl group of the pyrrolidine ring. Therefore, it is feasible that the preferred initial oxidation site is influenced by the protonation status of nicotine and hence the pH value of the microenvironment in which metabolism occurs. Further evidence of the relevance of this for the catalytic activity of CYP336A1 is illustrated by the assessment of its substrate profile. On the basis of our screening, the catalytic activity of this P450 is limited to alkaloids containing a basic nitrogen. Highest activity can be observed for pyridine and tropane alkaloids that are closely related from a biosynthetic perspective and appear in the same plant families (especially Solanaceae) (*56*), indicating an adaptation to cope with concurrent alkaloid chemotypes.

Previous studies have shown a functional overlap of phylogenetically related insect P450s for natural (*40*) and synthetic xenobiotics (*28*, *57*). Our phylogenetic and syntenic analyses of the evolution of *CYP336* genes in Hymenoptera revealed that representatives of this P450 family are found in all major hymenopteran lineages encompassing sawflies, wasps, ants, and bees. Furthermore, the CYP336 family appears to be specific to Hymenoptera forming a sister group to the remaining members of the CYP3 clan (CYP9 and CYP6 families). Our analyses also revealed remarkable conservation of the genes framing the CYP336 locus across the superfamilies, from sawflies to bees. Assuming random gene order, the probability of finding two orthologs next to each other in three genomes has been calculated as <4 × 10^−6^ (*58*). Together, these results suggests that there has been strong selection to maintain *CYP336* gene presence and architecture across the diversity of the Hymenoptera indicative of an important functional role in this order.

Our functional analysis of seven additional *CYP336* genes from four highly diverged hymenopteran species revealed a conserved capacity to metabolize alkaloids. We investigated enzymes from species with diverse interactions with plants, including pollination (bee species), leaf-cutting (*A. cephalotes* and *M. rotundata*), and parasitism (*X. alpigena*). While the honeybee is an example of a generalist species interacting with many different plants, the sawfly *X. alpigena* is highly specialized on *Pinus cembra* (*59*). Unexpectedly, CYP336J10 from *X. alpigena* was found to exhibit activity against a broad range of alkaloids including nicotine, a compound that is unlikely to be present in its host plant. This suggests that the metabolic capacity of CYP336 enzymes toward alkaloids is wide-ranging and goes beyond chemotypes typically encountered by specialized species. From an evolutionary point of view, this may lead to an adaptive advantage in the event that novel xenobiotic challenges are encountered, or it may buffer against the variation in PSM content and composition in different host plants (*17*). For those species that have more than one CYP336 gene, our analyses reveal distinct, but complementary, encoded substrate preferences of paralogous genes. This is consistent with the theory of (arthropod) P450 evolution where “the catalytic competence of a P450 may be viewed as a cloud of substrate structure space,” which can shift after a gene duplication event resulting in sub- or neofunctionalization (*60*). In support of this, we show that *CYP336A* isoforms in the bumblebee and alfalfa leaf-cutting bee are divergently expressed in different tissues or body segments consistent with regulatory sub- or neofunctionalization.

Our investigation of candidate amino acid residues that might be key to the conserved function of CYP336 enzymes across Hymenoptera revealed an amino acid, D298, which is highly conserved in CYP336 enzymes of hymenopteran lineages, but where the remaining CYP3 clan P450s usually has an alanine or glycine at the corresponding position. Our computational modeling suggested that D298 functions as a gatekeeper by facilitating access and binding of protonated substrates such as alkaloids within the catalytic pocket of CYP336 genes. Site-directed mutagenesis confirmed the importance of this residue for alkaloid metabolism by revealing a selective role of D298 as an “on/off”-switch for nicotine and atropine metabolism. A complete loss of function (here alkaloid degradation) following a single amino acid substitution is remarkable for an insect P450 (*29*). Furthermore, the observed gain of catalytic activity toward a nonbasic coumarin model substrate further supports the hypothesis that the aspartic acid residue in the I-helix serves as an important determinant for the selectivity of CYP336A1 toward protonated alkaloids in general.

In conclusion, our data reveal a conserved mechanism of PSM detoxification in hymenopteran species that diverged over 281 Ma (*42*) and resolve a key functional determinant of PSM metabolism at the amino acid level. These findings contribute to the understanding of PSM detoxification in beneficial insects and suggest that insects from the same order, but with contrasting lifestyles, recruit similar, conserved mechanisms to deal with potentially toxic PSM (*61*). These results provide fundamental insight into the molecular basis of adaptations arising from interactions between plants and beneficial insects and are also of applied importance in advancing our understanding of the molecular basis of the selectivity of insecticides from natural and synthetic origins.

## MATERIALS AND METHODS

### Insects

Honeybees (*A. mellifera* L.) were obtained from queen-right colonies located in Monheim am Rhein, Germany. Bumblebee colonies were purchased from Agralan Ltd. (Swindon, UK) and kept in constant darkness at 25°C, 50% relative humidity. The hives were fed ad libitum with the nectar substitute, Biogluc, and pollen was supplied to colonies every 2 days. Adult alfalfa leaf-cutting bee (*M. rotundata*) cocoons were obtained from Canada through Bayer AG Crop Science Division (Monheim, Germany) in 2021, brought to emergence as described previously (*62*), and subsequently frozen in liquid nitrogen and stored at −80°C until RNA isolation.

### Chemicals

All chemicals were of the highest purity available and obtained from Sigma-Aldrich (St. Louis, USA), if not stated otherwise. Investigated phytochemicals included nicotine (CAS 54-11-5) and its metabolite cotinine (CAS 486-56-6), atropine (CAS 51-55-8), anabasine (CAS 13078-04-1), scopolamine (CAS 51-34-3), echimidine (CAS 520-68-3), cytisine (CAS 485-35-8), coumarin (CAS 91-64-5), and caffeine (CAS 58-08-2). *N*-methylmyosmine (CAS 525-74-6) was custom-made and purchased from Carbosynth (Newbury, UK).

Model substrates included BFC (CAS 220001-53-6), 7-ethoxy-coumarin (EC; CAS 31005-02-4), 7-methoxy-coumarin (MC; CAS 531-59-9), 7-ethoxy-4-(trifluoromethyl)-coumarin (EFC; CAS 115453-82-2), 7-methoxy-4-(trifluoromethyl)-coumarin (MFC; CAS 575-04-2), 7-hydroxy-4-(trifluoromethyl)-coumarin (HFC; CAS 575-03-1), 7-benzyloxy-methoxy-resorufin (BOMR; CAS 87687-02-3), 7-ethoxy-resorufin (ER; CAS 5725-91-7), 7-benzyloxy-resorufin (BR; CAS 87687-02-3), 7-methoxy-resorufin (MR; CAS 5725-89-3), 7-pentoxy-resorufin (PR; CAS 87687-03-4), and *p*-nitroanisole (CAS 100-17-4).

7-Benzyloxy-methyloxy-4-(trifluoromethyl)-coumarin (BOMFC; CAS 277309-33-8) was custom-synthesized by Enamine Ltd. (Riga, Latvia), and 7-pentoxy-coumarin (PC) was synthesized in-house (Bayer AG, Monheim, Germany). BOMR was purchased from Invitrogen (Thermo Fisher Scientific, USA).

### In vitro screening of P450-mediated alkaloid metabolism

Functional expression of recombinant P450s was performed exactly as described previously (*26*, *27*). P450s were coexpressed with *A. mellifera* NADPH [reduced form of nicotinamide adenine dinucleotide phosphate (NADP^+^)]–dependent cytochrome P450 reductase using a Bac-to-Bac baculovirus expression system (Invitrogen) in insect High five cells (a clonal isolate derived from the ovary of cabbage looper, *T. ni*, purchased from Thermo Fisher Scientific). Site-directed mutagenesis of CYP336A1 was conducted using the Q5 Site-Directed Mutagenesis Kit following the manufacturer’s instructions (New England Biolabs Inc., USA) and with primers designed for the codon-optimized plasmid sequence (Fw: TTCTACCTGGGCGGTGTCGAG; Rv: GGACACGGCGTGAGCAGC).

The functional activity of recombinantly expressed microsomal P450s was tested against a range of fluorogenic coumarin- and resorufin-based model substrates as recently described (*63*). Activity against *p*-nitroanisole was determined according to Rose *et al.* (*64*) with slight modifications. Formation of the O-demethylation product *p*-nitrophenol was followed at 405 nm (bandwidth: 3.5 nm) in 15-s intervals for 15 min using the kinetic mode of the microtiter plate reader (Tecan Spark, Tecan Group, Männedorf, Switzerland).

Alkaloid depletion was assessed by incubating 80 μg of microsomal protein with 10 μM of the parent compound for 2 hours at 30°C. The 100-μl reactions included a NADPH regeneration system [Promega; 1.3 mM NADP^+^, 3.3 mM glucose-6-phosphate, 3.3 mM MgCl_2_, and glucose-6-phosphate dehydrogenase (0.4 U/ml)]. Control samples lacked the NADPH regeneration system. Additional controls contained microsomal fractions of insect cells coinfected with an empty baculovirus and *A. mellifera* cytochrome P450 reductase. The reaction was stopped with the addition of 400 μl of ice-cold acetonitrile, the samples were stored overnight at 4°C and centrifuged for 30 min at 3200*g*, and the supernatant was used for subsequent UPLC-MS/MS analysis.

Michaelis-Menten kinetic studies were conducted as follows: 20 μg of CYP336A1 or 160 μg of CYP6AQ1 was incubated for 30 min with different nicotine concentrations in twofold dilution steps ranging from 100 to 0.78 μM. All other factors remained the same.

Alkaloids were quantified by UPLC-MS/MS using Agilent 1290 Infinity II (Agilent Technologies, CA, USA) with a Waters Acquity HSS T3 column (2.1 × 50 mm, 1.8 mm) for all alkaloids except scopolamine (Acquity BEH, 2.1 × 50 mm, 1.8 mm column). Eluents (formic acid dissolved in acetonitrile or H_2_O) were used in gradient mode. After positive electrospray ionization, ion transitions were recorded on Sciex API6500 Triple Quad (AB Sciex LLC, MA, USA). All alkaloids were measured in positive ion mode (ion transitions: nicotine 163.1 > 130, anabasine 163.1 > 146.1, atropine 290.1 > 124.1, echimidine 398 > 119.9, scopolamine 304.1 > 138.1, cytisine 191.1 > 148.1, caffeine 195 > 138, coumarin 147 > 91). Peak integrals were calibrated against a standard calibration curve. Parent depletion was calculated by subtracting the values from +NADPH samples from the average of −NADPH replicates.

Nicotine metabolite quantification was conducted using purchased reference substances of cotinine and *N*-methylmyosmine. Before UPLC-MS/MS analysis (conducted as described above), the supernatant was purified using a SOLA-CX cartridge (Thermo Fisher Scientific, USA). Nicotine (15 μM) was incubated with recombinant CYP336A1 as described above. Sample (360 μl) was mixed with 2 ml of 5 mM ammonium formate (pH 2.5). The cartridge was conditioned with 500 μl of acetonitrile followed by 500 μl of 5 mM ammonium formate (pH 2.5) before sample application. The column was washed with 1 ml of 5 mM ammonium formate (pH 2.5) followed by 1 ml of 1% (v/v) formic acid in acetonitrile, evaporated under nitrogen at room temperature before the sample was eluted with 300 μl of 5% ammonia (v/v) in acetonitrile.

### Modeling of protein structure and in silico studies

A three-dimensional model of *A. mellifera* CYP336A1 was generated using the AlphaFold2 structure prediction software (*45*, *46*). The CYP336A1 primary sequence was submitted to the ColabFold web interface (*46*), and default options were selected to run AlphaFold2, with the multiple sequence alignment step performed using MMseqs2 (*65*). The quality and stereochemical soundness of the model was assessed using ERRAT (*66*) and PROCHECK (*67*), and Ramachadran plots were generated using the Structural Analysis and Verification Server (https://saves.mbi.ucla.edu/). The generated model lacked the heme group and so was aligned with CYP3A4 (PDB: 4D6Z) to transfer the heme group using the Schrodinger Maestro suite (Schrodinger, USA). The model was refined for further analysis by adding hydrogens, creating zero-order bonds to metals and disulfide bonds. The heme iron charge was set to +3, and the entire enzyme was energy-minimized.

Subsequently, the structure was uploaded to the CAVER Web 1.1. tool (*47*) for further analysis starting from the best hit for the catalytic pocket (relevance score, 100%; druggability score, 0.88). Tunnel detection was carried out considering all residues and with default settings. A transport analysis was conducted with protonated nicotine and the two relevant channels. Again, all residues were considered for the calculation of lower and upper bound energy limits.

### Similarity analysis

Molecular similarity analysis was performed using a simple python script within a Jupyter Notebook (see “Data and materials availability” statement for link to code). Key packages for the similarity analysis are RDKit (www.rdkit.org) for the actual similarity calculations and NetworkX (*68*) for visualization. Morgan Fingerprints (*69*, *70*), with a radius of 2 and as implemented in RDKit, were calculated for the investigated molecules, all in their neutral form. We experimented with Dice and Tanimoto metrics (*71*) and found that the Dice metric particularly suited for the problem at hand. We calculated the full similarity matrices and then created fully connected, undirected, and labeled graphs with the molecules as vertices and the respective similarities as edges. For the final graphs, all edges with a similarity value under a threshold (here: 0.3) were discarded, thus highlighting only the most relevant connections and similarity relationships. Thresholds other than 30% deliver different networks; however, the trends do not critically depend on the threshold.

### Quantum chemical calculations and Fukui functions

The calculations used a modified scheme as used previously (*33*), but the fundamentals remain the same (*72*). Molecular geometries of neutral and protonated species were prepared using Maestro 13.1 (Schrödinger LLC, USA). Quantum chemical calculations were carried out using the ORCA 5.0 suite (*73*, *74*). Visualization of Fukui functions was created with ChemCraft (https://chemcraftprog.com). Molecular geometries were optimized at BP86 level of theory (*75*, *76*) with an Ahlrichs def2-TZVP(-F) basis set (*77*). Electron densities were obtained by unrestricted single-point calculations at a slightly higher level of theory, B3LYP with def2-TZVP basis sets. We may denote these electron densities by ρ*_N_*(***r***). The electron densities of the oxidized and reduced species, ρ_*N*−1_(***r***) and ρ_*N*+1_(***r***), respectively, were calculated at the same level of theory. The Fukui functions were obtained numerically by the finite differences (*33*). All optimizations and single-point calculations used the CPCM (*78*) [with a COSMO (*79*) epsilon function] to mimic solvent effects in water. To better describe nonbonded weak interactions, Grimme’s D3 correction (*80*, *81*) was used throughout all calculations.

### Bioinformatic and phylogenetic analysis

Genomic and transcriptomic assemblies from hymenopteran species (Hymenoptera: taxid 7399) were retrieved from the NCBI database (www.ncbi.nlm.nih.gov/). Cytochrome P450s were identified using BLAST2GO (*82*) and InterProScan (*83*). CYP3 clan P450 from 48 species was selected on the basis of a BLASTN search with *A. mellifera* CYP3 clan P450s as query sequences (table S2). Protein sequences were aligned using the MUSCLE algorithm (*84*) within the Geneious software suite (Biomatters, New Zealand). MEGAX (*85*) was used to determine the best-fit model of amino acid substitution, using a maximum likelihood fit of 56 different models. Parameters including substitution model, proportion of invariable sites, and rate variation were calculated. The substitution model with the lowest Bayesian Information Criterion score was selected for use in phylogeny estimation. A maximum likelihood tree was created using PhyML (*86*) with LG+G (*87*) as the substitution model and 100 bootstraps. For the identification of subfamily determining positions (SDPs), the SDPlight algorithm (*43*, *88*) was used to identify amino acid residues differing significantly between CYP336 genes and remaining CYP3 P450s of selected hymenopteran species. The sequences encoding CYP336 P450s were then used in a second more focused phylogeny using Bayesian inference (*89*) [substitution model LG+G (*87*); chain length, 1,100,000; subsampling frequency, 200; burn-in length, 100,000; heated chains, 4; heated chain temperature, 0.2].

Analysis of macrosynteny was conducted as follows: Genomic sequences containing CYP336 sequences were retrieved from the NCBI database for *A. mellifera* (DH4 linkage group LG2, Amel_HAv3.1), *Ooceraea biroi* (chromosome 9, Obir_v5.4), *Solenopsis invicta* (chromosome 3, UNIL_Sinv_3.0), *Vespa crabo* (chromosome 25, iyVesCrab1.2), *V. canescens* (chromosome 1, ASM1945775v1), *Nasonia vitripennis* (chromosome 2, Nvit_psr_1.1), and *N. lecontei* (chromosome 6, iyNeoLeco1.1). Syntenic analyses between these chromosomes were performed using Mauve (multiple alignment of conserved genomic sequence with rearrangements) version 2.4.0 (*90*, *91*). This allowed for order and orientation of segments to be displayed and all LCBs to be outlined. To allow comparison of the number of LCBs in the pairwise alignments, the LCB weight was set at >400 to <550 bp.

To be able to evaluate both inter- and intra-superfamily microsynteny, a further five species were added to the analyses: *Athalia rosea* (iyAthRosa1.1), *Cotesia glomerata* (MPM_Cglom_v2.3), *Ceratosolen solmsi* (CerSol_1.0), *Polistes dominula* (Pdom r1.2), and *M. rotundata* (MROT_1.0) (table S2). The region ~500 kbp upstream and downstream of the CYP336 locus was examined in more detail for evidence of microsynteny. A series of inter- and intra-superfamily pairwise comparisons were run on these chromosomal extractions, using Mauve (*90*, *91*). The extracted genomic regions were examined manually, and all named flanking genes were noted. For a region to be considered a syntenic block, the minimum requirement was the conservation of two neighboring orthologs with no more than five unrelated genes in the intervening DNA.

### mRNA expression profiling of bee P450s

Expression of bee CYP336A P450s in body parts and dissected tissues was investigated by qPCR. Female bees were snap-frozen in liquid nitrogen and stored at −80°C until further use. Honeybees and bumblebees were transferred to RNA*later* ICE (Thermo Fisher Scientific, USA) and kept at −20°C for at least 24 hours, before the dissection of midgut, Malpighian tubules, and brain. Alfalfa leaf-cutting bees were directly divided into three segments (head, thorax, and abdomen) using forceps and scissors on dry ice. Subsequently, tissues were disrupted using 3-mm stainless steel beads in a bead mill at 20 Hz for 2 × 30 s. Total RNA was extracted using TRIzol reagent (Invitrogen, USA) following the manufacturer’s instructions. RNA purification was performed using the RNeasy Mini Kit (Qiagen, Germany) for *M. rotundata* samples and the PicoPure RNA isolation kit (Thermo Fisher Scientific, USA) for *A. mellifera* and *B. terrestris* tissue samples. RNA was eluted in appropriate volumes of nuclease-free water.

RNA quality was assessed using the QIAxcel RNA QC Kit v2.0 (Qiagen, Germany). One microgram was used for complementary DNA (cDNA) synthesis using the iScript cDNA Synthesis Kit (Bio-Rad, USA) according to the manufacturer. For tissue samples from *A. mellifera* and *B. terrestris*, PCRs (10 μl) contained 0.5 ng of cDNA, 5 μl of SsoAdvanced Universal SYBR Green Supermix (Bio-Rad, USA), and 0.25 μM of each primer. Samples were run on the CFX 384 Real-Time System (Bio-Rad, USA) under the following conditions: 3 min at 95°C followed by 39 cycles of 95°C for 15 s, 64°C for 15 s, and 60°C for 15 s. *M. rotundata* samples were run under slightly different conditions (2 ng of cDNA template; thermal cycling conditions: 3 min at 95°C and 40 cycles of 95°C for 15 s and 60°C for 30 s). A final melt-curve step was included after PCR (ramping from 65° to 95°C by 0.5°C every 5 s) to confirm the absence of any nonspecific amplification in all cases. The efficiency of PCR for each primer pair was assessed using a fivefold serial dilution of cDNA. Each reverse transcription–qPCR experiment consisted of at least four independent biological replicates with three technical replicates. Data were analyzed by qbase+ version 3.1 (Biogazelle, Belgium). For honeybees, the reference genes Rpl32 (ribosomal protein L32), EF1a (elongation factor 1α), and RPS5 were used (*92*). For bumblebees, phospholipase A2, arginine kinase, and EEF1A served as reference genes (*93*). For alfalfa leaf-cutting bees, RPL8 and RPL27A were used. All reference genes were tested for their stability before selection. The sequences of all primers used in qPCR are detailed in table S7.

## Notes

View/request a protocol for this paper from *Bio-protocol*.
