# Supplementary material for: A conserved hymenopteran-specific family of cytochrome P450s protects bee pollinators from toxic nectar alkaloids
Source: Sci Adv. 2023 Apr 12;9(15):eadg0885. doi: 10.1126/sciadv.adg0885 (PMC10096648; doi:10.1126/sciadv.adg0885)
Supplement: Supplementary file 1 — Figs. S1 to S9 Tables S1 to S7 Legend for data S1 [file sciadv.adg0885_sm.pdf]

Science Advances
AAAS

Supplementary Materials for

A conserved hymenopteran-specific family of cytochrome P450s protects bee pollinators from toxic nectar alkaloids

Julian Haas *et al.*

Corresponding author: Chris Bass, c.bass@exeter.ac.uk; Ralf Nauen, ralf.nauen@bayer.com

Sci. Adv. **9**, eadg0885 (2023)
DOI: 10.1126/sciadv.adg0885

The PDF file includes:

Figs. S1 to S9
Tables S1 to S7
Legend for data S1

Other Supplementary Material for this manuscript includes the following:

Data S1

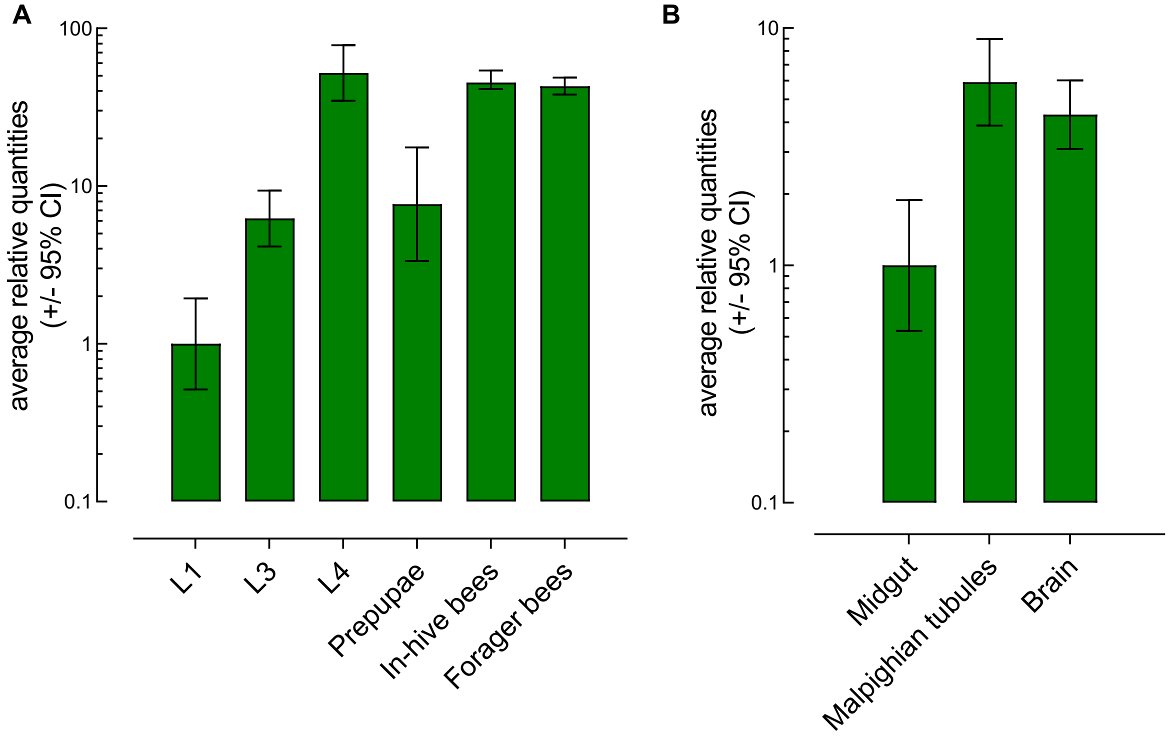

Figure S1. Relative expression of CYP336A1 in different (A) life stages and (B) tissues of the honey bee *A. mellifera* as measured by quantitative PCR. Error bars display 95% confidence intervals (n = 4).

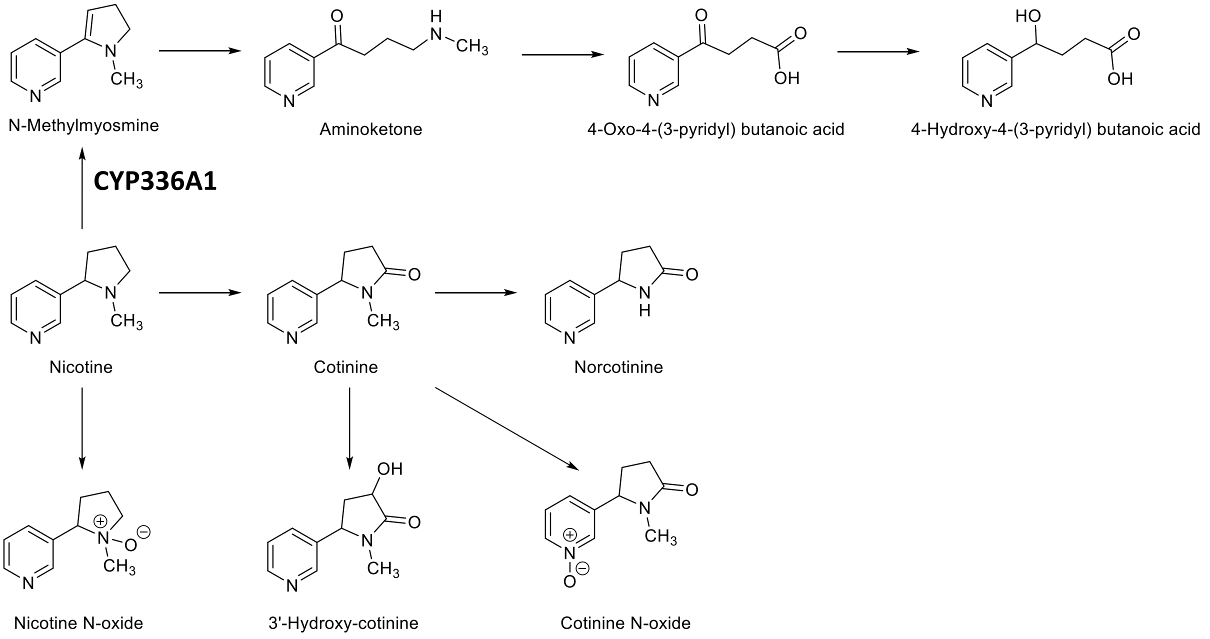

Figure S2. Potential metabolic fate of nicotine in honeybees according to du Rand et al. (ref[24]) with the insertion of *N*-methylmyosmine as the first step of the C'2 oxidation pathway in honeybees and the major product of CYP336A1-mediated metabolism.

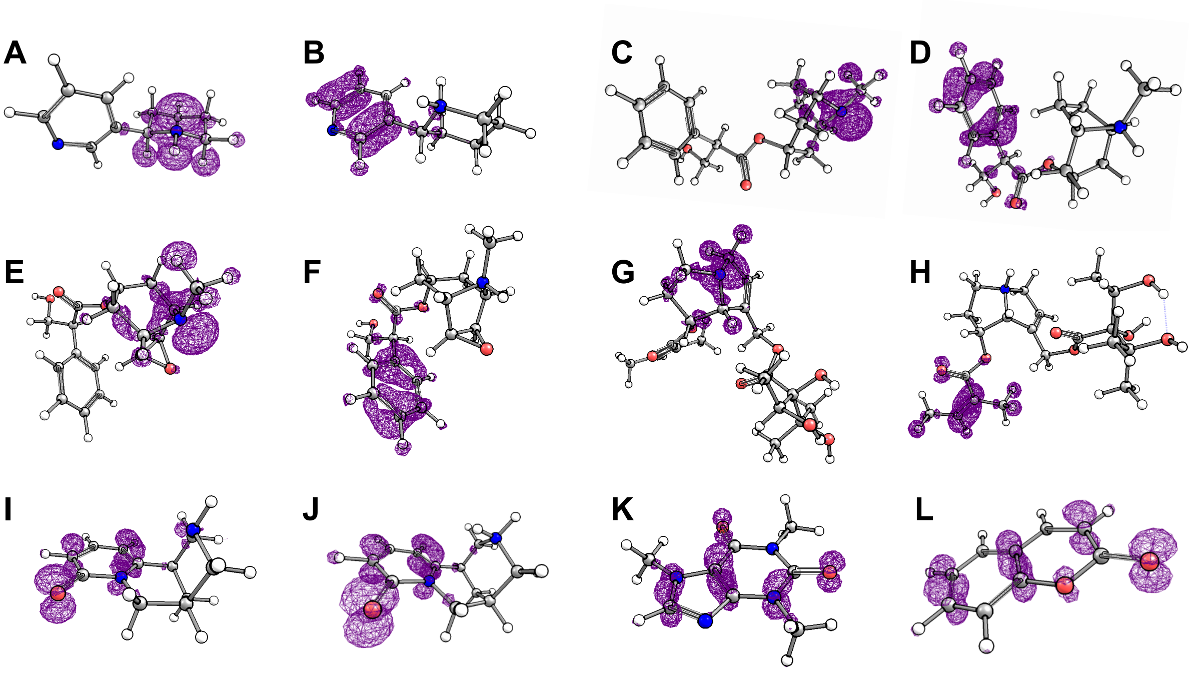

Figure S3. Fukui functions for attack by an electrophile ($f^{-}(r)$) of investigated phytochemicals. The contours are at 0.005 a.u. For more information, see methods. (A) deprotonated anabasine; (B) protonated anabasine; (C) deprotonated atropine; (D) protonated atropine; (E) deprotonated scopolamine; (F) protonated scopolamine; (G) deprotonated echimidine; (H) protonated echimidine; (I) deprotonated cytisine; (J) protonated cytisine; (K) caffeine; (L) coumarin.

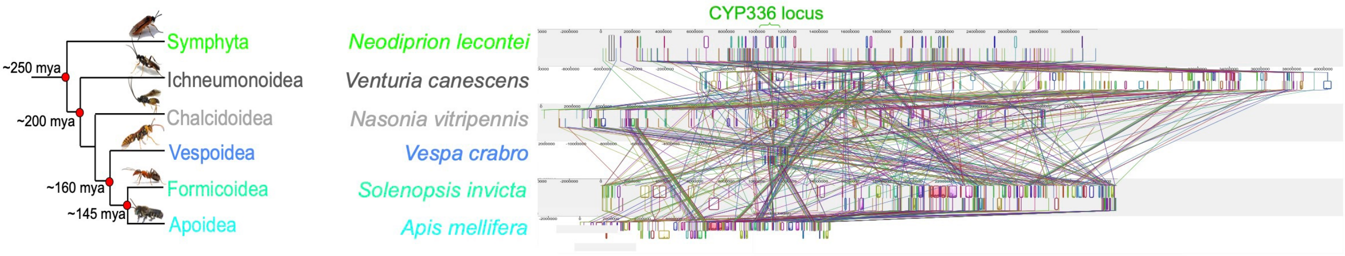

Figure S4: Macro-syntenic relationship of chromosomes containing the CYP336 loci in six hymenopteran species from six superfamilies. Each coloured shape is a region without rearrangement of homologous backbone sequence (a collinear block). Lines between sequences trace orthologous LCBs through the genomes.

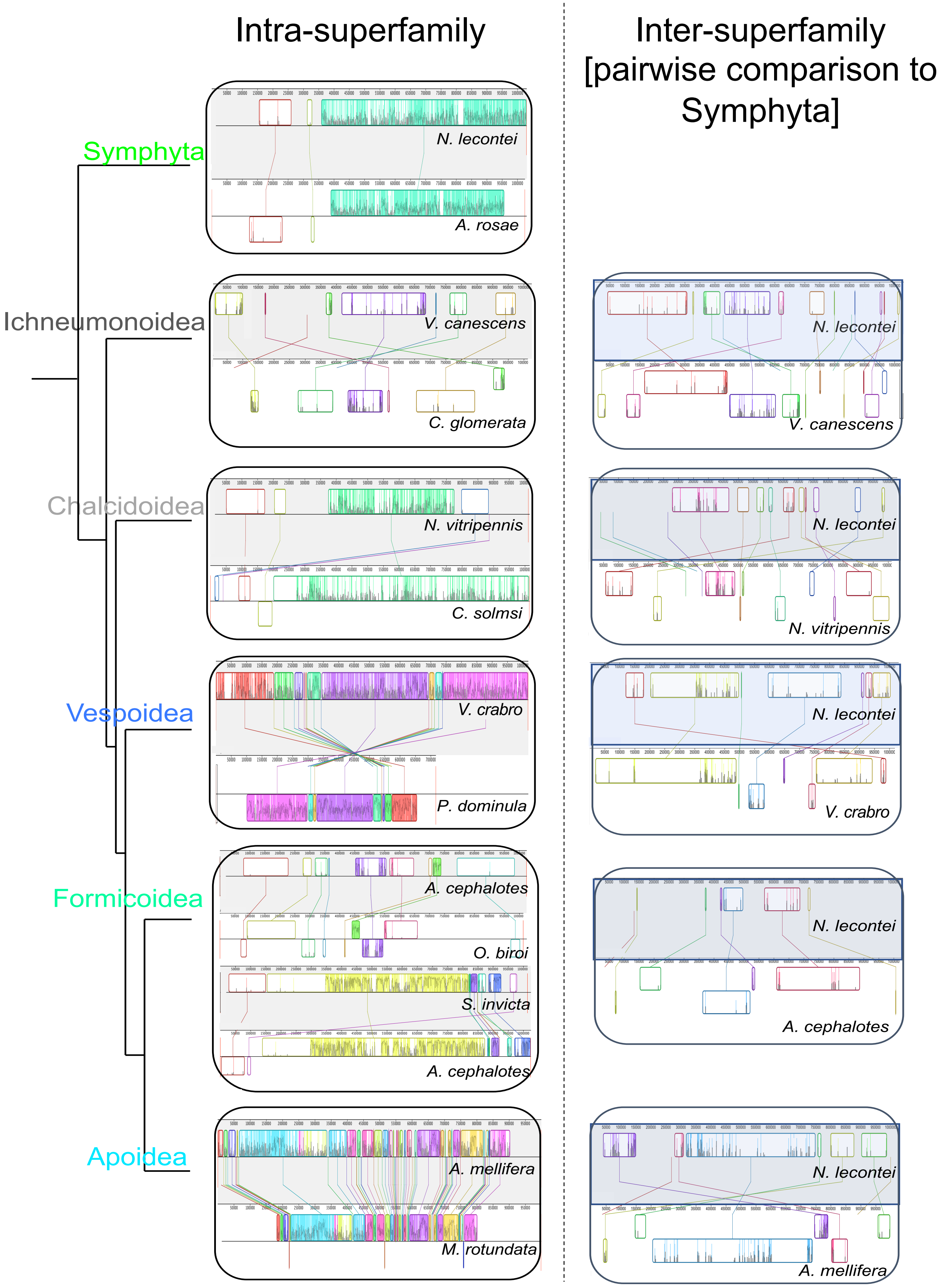

Figure S5: Pairwise comparison of intra- and inter-superfamily micro-synteny at the CYP336 loci in hymenopteran species from six superfamilies. Each coloured shape is a region without rearrangement of homologous backbone sequence (a collinear block). Lines between sequences trace orthologous LCBs through the genomes.

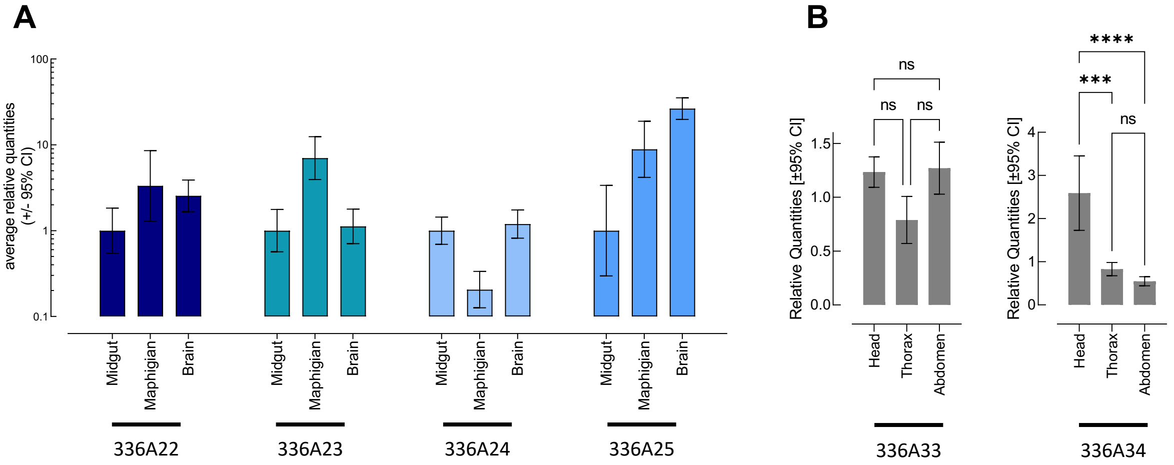

Figure S6. Expression pattern of CYP336A-orthologs measured via qPCR. (A) Relative expression of bumble bee *CYP336A* genes in midgut, Malphigian tubules and brain. (B) Relative expression of alfalfa leafcutting bee *CYP336A* genes in three body segments (head, throax, abdomen).

PROCHECK

Ramachandran Plot

saves

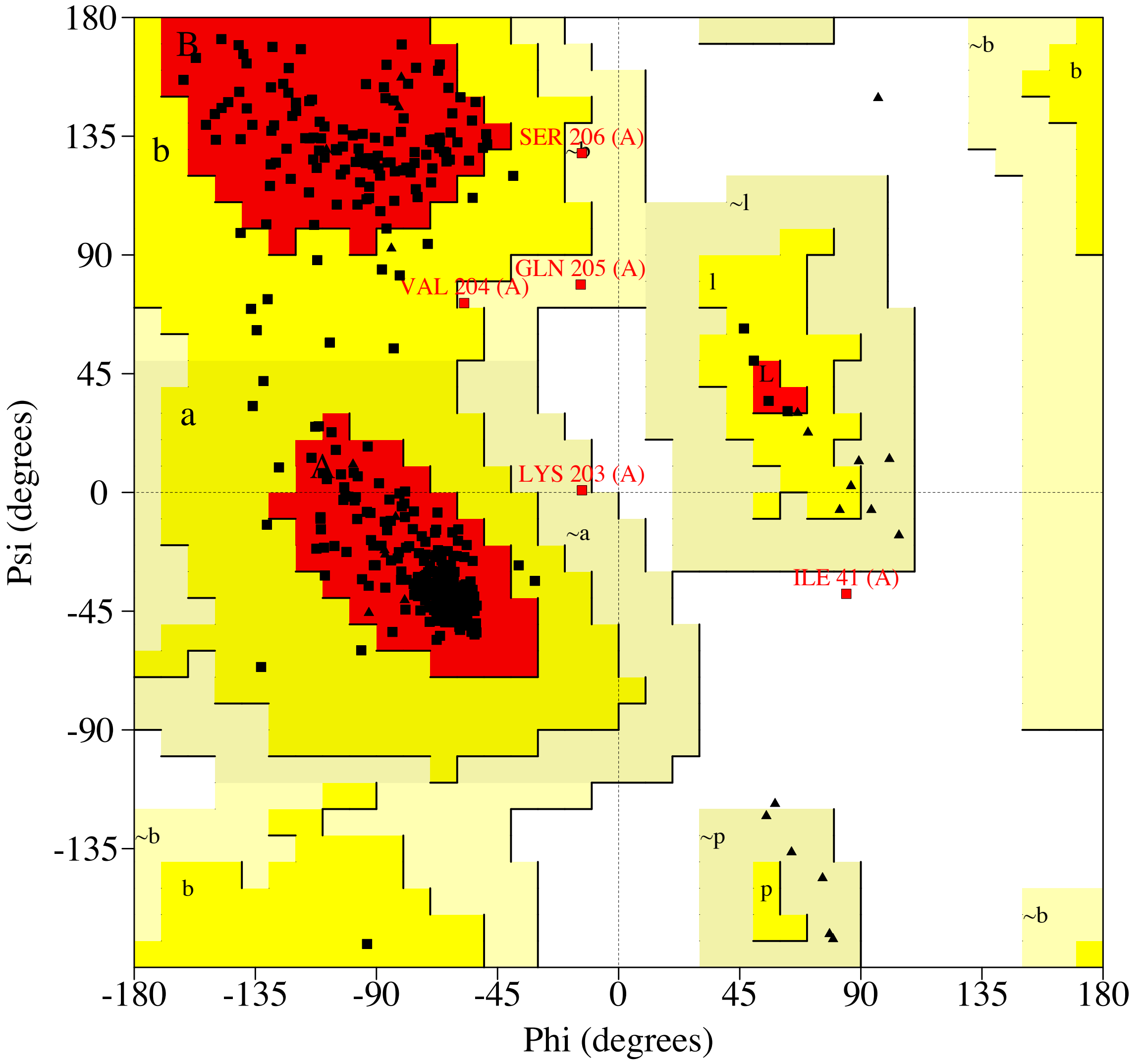

Plot statistics

Residues in most favoured regions [A,B,L]	414	93.5%
Residues in additional allowed regions [a,b,l,p]	24	5.4%
Residues in generously allowed regions [~a,~b,~l,~p]	4	0.9%
Residues in disallowed regions	1	0.2%
	----	------
Number of non-glycine and non-proline residues	443	100.0%
Number of end-residues (excl. Gly and Pro)	2	
Number of glycine residues (shown as triangles)	29	
Number of proline residues	22	

Total number of residues	496	

Based on an analysis of 118 structures of resolution of at least 2.0 Angstroms and R-factor no greater than 20%, a good quality model would be expected to have over 90% in the most favoured regions.

Figure S7. Ramachandran plot of the *A. mellifera* CYP336A1 protein model.

Tunnels

Tunnel	Bottleneck radius [A]	Length [A]	Curvature	Throughput
1	1.4	13.3	1.2	0.7
3	1.1	20.0	1.3	0.4

Tunnel – identifier of protein tunnel; *Bottleneck radius* – radius of the narrowest part of the tunnel; *Length* – length of the tunnel; *Curvature* – the curvature of the tunnel; *Throughtput* – troughput of the tunnel.

CaverDock results

Job ID	Ligand	Tunnel	Direction	E_{Bound} [kcal/mol]	E_{Max} [kcal/mol]	$E_{Surface}$ [kcal/mol]	E_a [kcal/mol]	ΔE_{BS} [kcal/mol]
kr9jdlo0g8	NCT	3	IN	−6.6	21.7	2.0	19.7	−8.6
fxwwkmzdwv	NCT	1	IN	−6.7	−3.5	−4.5	1.0	−2.2

Tunnel – selected protein tunnel; *Ligand* – name of the used molecule; *Direction* – direction of the CaverDock calculation; E_{Bound} – the binding energy of the ligand located in the binding site; E_{Max} – a the highest binding energy in the trajectory; $E_{Surface}$ – the binding energy of the ligand lo at the protein surface; E_a – activation energy of association, E_{Max} - E_{Bound} for products, E_{Max} - $E_{Surface}$ for reactants (describes the difficulty of getting through the tunnel; kinetics); ΔE_{BS} – difference of the binding energies of the ligand in the active site and at the surface (corresponds to enthalpy; thermodynamics).

Figure S8. CaverDock analysis of protonated nicotine.

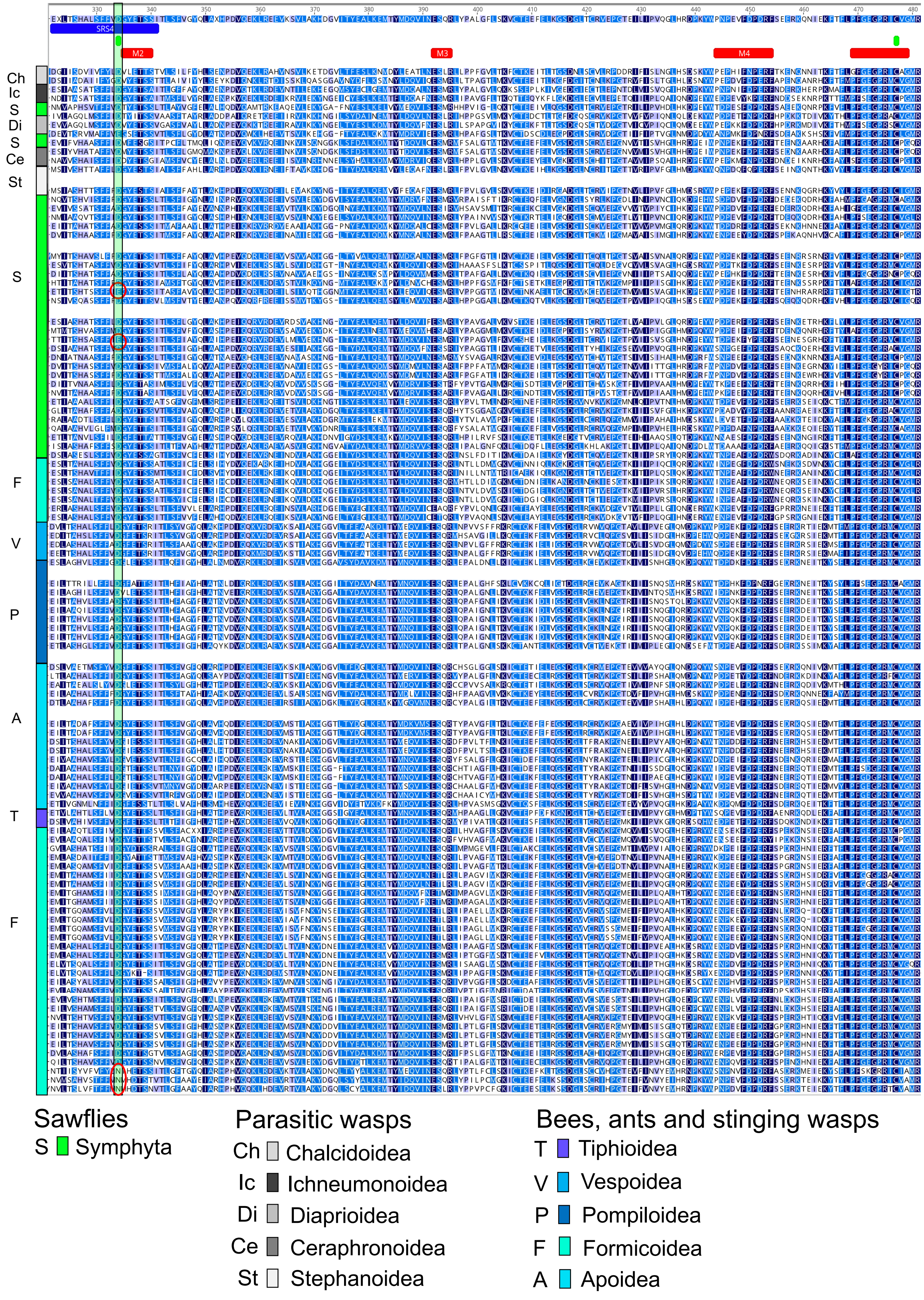

Figure S9. Multiple sequence alignment of CYP336 protein sequences of hymenopteran species from six superfamilies. Conserved motifs (M) 2-5 and substrate recognition site (SRS) 4 are annotated using red and blue. The conserved aspartic acid residue (D) is highlighted in green. Exceptional residues are outlined in red – sawflies *Athalia rosae* and *Xiphydria camelus* D/E substitution and the ant *Dinoponera quadriceps* three sequences D/N.

Table S1. Model substrate activity of investigated P450s in pmol product per min and mg protein (n = 4). Values for p-nitroanisole are in A_{405}/min * mg protein. CYP9Q3 coumarin derivatives measurements are taken from Haas et al. (ref[28]). n.d. = not determined

		Coumarin derivatives													
		BFC		BOMFC		EFC		MFC		EC		MC		PC	
Organism	P450	Mean	SD	Mean	SD	Mean	SD	Mean	SD	Mean	SD	Mean	SD	Mean	SD
A. mellifera	CYP9Q3	54.43	1.11	100.38	3.92	11.89	0.33	2.41	0.07	12.25	0.37	n.d.	n.d.	6.24	0.89
A. mellifera	CYP336A1	0.00	0.00	0.00	0.00	0.00	0.00	0.00	0.00	0.00	0.00	0.04	0.03	0.00	0.00
R. norvegicus	Liver microsomes	n.d.	n.d.	n.d.	n.d.	n.d.	n.d.	n.d.	n.d.	n.d.	n.d.	n.d.	n.d.	n.d.	n.d.

		Resorufin derivativies										Anisoles	
		BOMR		BR		PR		ER		MR		P-Nitroanisole	
Organism	P450	Mean	SD	Mean	SD	Mean	SD	Mean	SD	Mean	SD	Mean	SD
A. mellifera	CYP9Q3	98.60	2.88	10.62	1.63	0.66	0.30	1.67	0.76	0.00	0.00	n.d.	n.d.
A. mellifera	CYP336A1	0.63	0.82	0.07	0.06	0.06	0.6	0.223	0.56	0.00	0.00	0.08	0.09
R. norvegicus	Liver microsomes	n.d.	n.d.	n.d.	n.d.	n.d.	n.d.	n.d.	n.d.	n.d.	n.d.	1.93	0.02

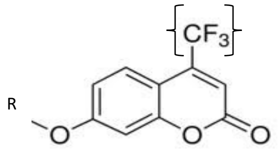

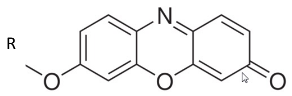

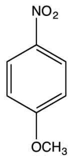

Table S2. List of hymenopteran species included in the phylogenetic and syntenic analyses of the P450 clade 3 and CYP336 family. WGS = Whole Genome Sequencing; TSA = Transcriptome Shotgun Assembly; N/F = not found (incomplete assembly).

Species	WGS	TSA	Total Clan 3	CYP336
Acantholyda hieroglyphica		GBKM01	11	1
Apis mellifera	Amel_HAv3.1		30	1
Aproceros leucopoda		GBTP01	10	1
Arge berberidis		GBPH01	16	1
Athalia rosae	iyAthRosa1.1		24	5
Atta cephalotes	Attacep 1.0		28	5
Atta colombica	Acol 1.0		24	5
Blasticotoma filiceti		GBVF01	8	1
Bombus terrestris	Bter_1.0		32	4
Cephus cinctus	Ccin1		16	N/F
Ceratosolen solmsi	CerSol_1.0		9	1
Cimbex rubida		GBOL00000000.1	10	1
Colletes cunicularius		GBOL01	25	4
Cotesia glomerata	MPM_Cglom_v2.3		26	2
Dasymutilla gloriosa		GBOQ01	17	8
Dendrocerus carpenteri		GBOS01		2
Dinoponera quadriceps	ASM131382v1		54	9
Diodontus minutus		GBMA01	18	1
Diprion pini		GBUO01	11	1
Dolichurus corniculus		GBUO01	42	4
Dufourea novaeangliae	ASM127255v1		28	4
Fopius arsianus	ASM80636v1		15	N/F
Habropoda laboriosa	ASM126327v1		21	2
Lasius niger	ASM104565v1		48	5
Megachile rotundata	MROT_1.0		30	3
Megalodontes cephalotes		GBQG01	28	2
Monosapyga clavicornis		PRJNA252301		1
Nasonia vitripennis	Nvit_psr_1.1		55	1
Nematus ribesii		GBQG01	13	1
Neodiprion lecontei	iyNeoLeco1.1		33	3
Ooceraea biroi	Obir_v5.4		42	6
Orussus abietinus	Oabi_2.0		12	1
Pepsis grossa		GAXS01	61	5
Pergagrapta polita		GBWZ01	10	1
Polistes dominula	Pdom r1.2		17	2
Pseudomasaris vespoides		GAXQ01	12	1
Pseudomyrmex gracilis			50	4
Scolia hirta		GBQJ01	14	1
*Sphex funerariu*s		GBQD01	13	1
Stephanus serrator		GBMU01	26	4
Solenopsis invicta	UNIL_Sinv_3.0		90	6
Tenthredo koehleri		GAWW02	19	2
Tiphia femorata		GBWN01	21	3
Tremex magus		GBLX01	7	N/F
Trichopria drosophilae		GBUQ01	25	3
Venturia canescens	ASM1945775v1		26	2
Vespa crabro	iyVesCrab1.2		21	3
Vespa mandarinia	V.mandarinia_Nanaimo_p1.0		21	4
Xyela alpigena		GADA01	17	1
Xiphydria camelus		SRX642996	15	2

Table S3. Macro-synteny of the genomic region containing *CYP336A* sequences as determined by number of locally colinear blocks (LCBs) in pairwise comparisons using *N. lecontei* chromosome 6 as a reference sequence.

Reference (Symphyta)	Query	LCB weight (bp)	No of LCBs
Neodiprion lecontei Chr 6 (~31.7 Mbp)	*Venturia canescens* Chr 1 (~41.1 Mbp) Ichneumonoidae	474	100
Neodiprion lecontei Chr 6 (~31.7 Mbp)	*Nasonia vitripennis* Chr 2 (~35.4 Mbp) Chalcidoidea	511	71
Neodiprion lecontei Chr 6 (~31.7 Mbp)	*Vespa crabo* Chr 25 (~3.6Mbp) Vespoidea	443	17
Neodiprion lecontei Chr 6 (~31.7 Mbp)	*Ooceraea biroi* Chr 9 (~15.6 Mbp) Formicoidea	406	57
Neodiprion lecontei Chr 6 (~31.7 Mbp)	*Solenopsis invicta* Chr 3 (~33 Mbp) Formicoidea	400	68
Neodiprion lecontei Chr 6 (~31.7 Mbp)	*Apis mellifera* LG2 (~16 Mbp) Apoidea	494	59

Table S4. Recombinantly expressed P450s and their NCBI accession number.

Species	Recombinant P450s	NCBI Accession number	Residue 103	Residue 119	Residue 298	Residue 369	Residue 407
A. mellifera	CYP336A1	XP_001119981.4	N	P	D	K	W
B. terrestris	CYP336A22	XP_020723943.1	N	P	D	K	W
	CYP336A23	XP_003393865.1	N	P	D	K	W
	CYP336A24	XP_003393867.1	N	P	D	K	W
M. rotundata	CYP336A33	XP_003702293.1	N	P	D	K	W
	CYP336A34	XP_003702219.1	T	P	D	R	W
A. cephalotes	CYP336A60	XP_012057979.1	N	P	D	K	W
X. alpigena	CYP336J10	GBVH01014829.1	N	L	D	K	W

Table S5. Average depletion [%] of tested alkaloids by recombinantly expressed P450s.

	CYP336A1	CYP336A33	CYP336A34	CYP336A60	CYP336J10	CYP336A22	CYP336A23	CYP336A24
**Nicotine**	100.00	15.27	100.00	90.46	100.00	92.15	39.83	48.26
**Anabasine**	79.75	44.90	100.00	62.86	66.36	41.05	6.43	99.97
**Atropine**	100.00	55.14	70.50	75.73	96.78	59.34	94.10	84.72
**Cytisine**	24.01	26.81	9.51	26.32	22.97	33.60	33.88	37.74
**Scopolamine**	38.75	0.37	0.00	16.55	100.00	12.94	70.20	2.38
**Echimidine**	21.38	82.57	0.00	76.60	87.12	n.d.	n.d.	n.d.

Table S6. Amino acid residues identified as significantly different between CYP336 enzymes and remaining CYP3 clan P450s of 40 investigated hymenopteran species by the SDPlight algorithm (Kalinina et al. ref[43]). The position of substrate recognition sites (SRS) are annotated based on Gotoh et al. (ref[44]).

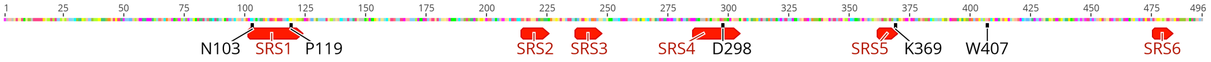

Residue CYP336A1 *Apis mellifera*	Residue in CYP336 family	Residue remaining Clade 3	Z-score
N103	NT	HRD	48.27
P119	PLA	LIVM	46.54
D298	DNE	AGS	47.14
K369	KR	RH	66.84
W407	W	YFWH	53.62

Table S7. Sequence of oligonucleotide primers used to assess the expression of CYP336A P450 genes in three bee species.

Target gene	Forward primer sequence	Reverse primer sequence
A. mellifera		
RPL32	AGTAAATTAAAGAGAAACTGGCGTAA	TAAAACTTCCAGTTCCTTGACATTAT
EF1a	GGAGATGCTGCCATCGTTAT	CAGCAGCGTCCTTGAAAGTT
RPS5	AATTATTTGGTCGCTGGAATTG	TAACGTCCAGCAGAATGTGGTA
CYP336A1	AAATCCTTTTCGCAGCCGTG	AGCCTTCGATTCCTAAGCCG
B. terrestris		
PLA2	GGTCACACCGAAACCAGATT	TCGCAACACTTCGTCATTTC
AK	TGTCGGTATCTACGCGCCTG	TTGGTGGATGCTTGTCAGTC
EEF1A	AGAATGGACAAACCCGTGAG	CACAAATGCTACCGCAACAG
CYP336A22	TCCTGCCTAAACACGTGGAT	GCTGCTACAATCTCTTCGGC
CYP336A23	TGGCTGAACGGAAGAAAACG	TCATCCGCGAAACAATGACC
CYP336A24	GGCCACCTGTTACCCCTAAT	GCAGCACGGTTTTCACTACA
CYP336A25	GGTTCTTTTCGCAGCTGTCA	TCATCCGCGAAACAATGACC
M. rotundata		
RPL8	CATCCAACAACAGGGCC	GCTCCACAGGGTTCATAGCA
RPL27A	GGGGACTGCGATCAAGATGT	AGTTGATACGGTGGTGGTGC
CYP336A33	CGAGCTGGAAGGATCAGACG	TTTTGCTTCCTGTCCTCGCT
CYP336A34	AAGAAGCGATCACAGCCGAA	AAACGTCAGCACCCCATCAT

Data S1. Genes flanking CYP336 P450s in select hymenopteran species (see associated Excel file). The gene order and direction is shown for each species.
